# A stillbirth calculator: Development and internal validation of a clinical prediction model to quantify stillbirth risk

**DOI:** 10.1371/journal.pone.0173461

**Published:** 2017-03-07

**Authors:** Amanda S. Trudell, Methodius G. Tuuli, Graham A. Colditz, George A. Macones, Anthony O. Odibo

**Affiliations:** 1 Mercy Hospital St. Louis Department of Obstetrics and Gynecology, Midwest Maternal Fetal Medicine, St. Louis, Missouri, United States of America; 2 Washington University School of Medicine Department of Obstetrics and Gynecology Division of Maternal Fetal Medicine, St. Louis, Missouri, United States of America; 3 Washington University School of Medicine Department of Surgery Division of Public Health, St. Louis, Missouri, United States of America; 4 University of South Florida, Moorsani College of Medicine Department of Obstetrics and Gynecology Division of Maternal Fetal Medicine, Tampa, Florida, United States of America; UNITED STATES

## Abstract

**Objective:**

To generate a clinical prediction tool for stillbirth that combines maternal risk factors to provide an evidence based approach for the identification of women who will benefit most from antenatal testing for stillbirth prevention.

**Design:**

Retrospective cohort study

**Setting:**

Midwestern United States quaternary referral center

**Population:**

Singleton pregnancies undergoing second trimester anatomic survey from 1999–2009. Pregnancies with incomplete follow-up were excluded.

**Methods:**

Candidate predictors were identified from the literature and univariate analysis. Backward stepwise logistic regression with statistical comparison of model discrimination, calibration and clinical performance was used to generate final models for the prediction of stillbirth. Internal validation was performed using bootstrapping with 1,000 repetitions. A stillbirth risk calculator and stillbirth risk score were developed for the prediction of stillbirth at or beyond 32 weeks excluding fetal anomalies and aneuploidy. Statistical and clinical cut-points were identified and the tools compared using the Integrated Discrimination Improvement.

**Main outcome measures:**

Antepartum stillbirth

**Results:**

64,173 women met inclusion criteria. The final stillbirth risk calculator and score included maternal age, black race, nulliparity, body mass index, smoking, chronic hypertension and pre-gestational diabetes. The stillbirth calculator and simple risk score demonstrated modest discrimination but clinically significant performance with no difference in overall performance between the tools [(AUC 0.66 95% CI 0.60–0.72) and (AUC 0.64 95% CI 0.58–0.70), (p = 0.25)].

**Conclusion:**

A stillbirth risk score was developed incorporating maternal risk factors easily ascertained during prenatal care to determine an individual woman’s risk for stillbirth and provide an evidenced based approach to the initiation of antenatal testing for the prediction and prevention of stillbirth.

## Introduction

In the United States (U.S.) 1/200 pregnancies reaching 22 weeks gestation will result in stillbirth[1]. Reduction in fetal mortality has been identified by the U.S. Department of Health and Human Services as an important goal which we have thus far failed to achieve. In Healthy People 2020, a goal for stillbirth reduction from 6.2/1,000 to 5.6/1,000 was scaled back after the original 2010 goal of 4.1/1,000 was not achieved[2]. Rates of stillbirth vary greatly among high income countries and the U.S. falls within the middle tier, indicating that as a country, we have room for improvement[1, 3].

In 2011, a series on stillbirth was published that addressed gaps and goals in stillbirth education and research which included improving antenatal screening for risk factors for stillbirth[4]. Several maternal characteristics assessed during prenatal care have been demonstrated to contribute to increased rates of stillbirth individually such as race, medical comorbidities, obesity and age[3]. Many characteristics such as chronic renal disease, or lupus, are considered to be high risk and warrant routine antenatal testing individually. However, many of these risk factors, such as black race, associated with two-fold odds for stillbirth, are considered minor, and most physicians would not recommend antenatal testing for such minor risk factors[5]. Currently, there is no widely accepted method that combines individual maternal characteristics to provide a patient specific stillbirth risk summary. Therefore, we aimed to generate a clinical prediction tool for stillbirth that combines individual maternal risk factors to provide an evidence based approach to the estimation of stillbirth risk with the goal of identifying women who may benefit most from antenatal testing but would otherwise not have met generally accepted indications for testing.

## Methods

The Washington University School of Medicine perinatal database was utilized. This previously validated database consists of prospectively collected information on singleton pregnancies presenting for routine second trimester anatomic screening from 1999–2009[6]. The study was approved by the Washington University Institutional Review Board June 18, 2013 as Project ID 20130633. Washington University is a quaternary referral center for a large catchment area in the Midwest including the entire state of Missouri, the Southern half of Illinois and Indiana, Western Tennessee and Kentucky, and Northeast Arkansas. Information regarding maternal sociodemographic, obstetric, maternal medical, genetic testing results and neonatal outcomes are systematically collected through the efforts of a dedicated research coordinator. At the time of inclusion, patients are given a survey to return following delivery and receive a phone call from the research coordinator if the survey has not been returned within four weeks of expected date of delivery. If the patient is unable to be contacted, the primary obstetrician is contacted for follow-up. Outcomes data are also gathered from the electronic medical record if delivery occurred within our healthcare system. There is over 90% compliance rate with survey return, therefore missing outcomes data is limited to the proportion of missing surveys that are subsequently lost to follow-up from their primary obstetrician’s office, or incomplete surveys that are unable to be completed from outside medical records. After exclusion of 12,280 with missing delivery information, a cohort of 64,173 was included for analysis. Sensitivity analysis has been previously reported demonstrating no difference in demographic characteristics of women excluded for lack of data or outcome information[6]. For individual model development, patients with missing predictor data were excluded. BMI was the predictor with the greatest number of missing data at <10%. When other predictors were missing data, these individual patients overlapped with those missing BMI and therefore did not contribute to any additional missing cases.

Logistic regression was used to develop models for the prediction of stillbirth. Variables considered as maternal risk factors for stillbirth were identified from the literature in combination with univariate analysis for stillbirth at or beyond 20 weeks gestation[3]. Starting with the most comprehensive model including all maternal risk factors, a backward stepwise selection process was utilized. Variables with non-significant p-values (>0.05) were identified first and elimination began with the variable with an odds ratio (OR) closest to 1. A variable was considered significant and kept in the model if there was a reduction in the discriminative ability of the model as determined by the area under the receiver operating characteristics curve (AUC) or if the AUC did not change but the beta-coefficients for the remaining variables changed by greater than 10%. If a continuous variable was found to have a significant impact on the model then it was further explored in various categorical and dichotomous forms. The final variable format used in the model was determined by the rules stated above for retention of significant variables.

At initial model development multiple models were evaluated for the prediction of stillbirth at or beyond the gestational ages of 20 weeks, 24 weeks, 28 weeks and 32 weeks, to generally explore model discrimination for the prediction of stillbirth throughout gestation. Each model excluded pregnancies ending prior to the gestational age specified. Additionally, to explore the role of anomalies and aneuploidy on the risk of stillbirth and stillbirth prediction, separate models were developed which either included fetal anomalies and aneuploidy as a risk factor or excluded anomalies and aneuploidy from the cohort.

In line with our clinically centered goal of identifying women who may benefit from antenatal testing, at this point in the investigation we moved forward with the model that predicts stillbirth at or beyond 32 weeks as The American College of Obstetricians and Gynecologists recommends 32 weeks for the initiation of antenatal testing for most patients[7]. Additionally, because fetal anomalies and aneuploidy could not be stratified by severity and this would be expected to impact the clinical decision for antenatal testing, we thought it more clinically applicable to exclude fetal anomalies and aneuploidy moving forward. When model discrimination appeared to be improved by an increase in the AUC but was not demonstrated to be significantly different, the beta coefficients were compared between models for changes of greater than 10% and model calibration was explored by evaluating the observed and expected events for centiles of probability. The Integrated Discrimination Improvement (IDI) was utilized to determine if there was a significant difference in model performance as described by Pencina et al.[8] Internal validation was carried out using bootstrapping with 1,000 repetitions and the 95% CIs surrounding the AUC are reported derived from the bootstrapped sample.

Our next step was to establish a clinically relevant probability cut-point that could be utilized to determine patients assigned to antenatal testing. The sensitivities and specificities were examined over a range of clinically relevant probabilities. Statistical cut-points were determined using the Youden Index and Liu test[9–11]. Clinical cut-points were explored through multiple case scenarios with the use of a stillbirth risk prediction calculator that was generated using the beta-coefficients from the final model.

To further simplify the model for bedside clinical use, a risk score was developed based on the odds-ratios for each covariate in the model. Estimated weights were determined by rounding the ORs to the nearest whole number which could then be summed to predict the final score for an individual patient based on the presence or absence of the risk factor. The AUC of the stillbirth risk score was then compared to the AUC of the multivariable model using the nonparametric method of comparison described by DeLong et al.[12]. The performance of the stillbirth calculator and the stillbirth risk score were compared clinically and statistically using the IDI. Finally, to address the increasing risk of stillbirth with advancing gestational age as demonstrated by several authors, models estimating the stillbirth risk at clinically appropriate gestational ages beyond 32 weeks were developed and compared[13–15].

Descriptive statistics were used to calculate maternal and pregnancy characteristics of the cohorts including and excluding fetal anomalies and aneuploidy. Univariate logistic regression was used to determine the odds-ratios with 95% confidence intervals for maternal characteristics used as candidate predictors. Tests of significance were determined at an α-level 0.05. Statistical analysis was performed using STATA software. *(version 12; StataCorp*, *College Station*, *TX)*

## Results

Of the 64,173 women who met inclusion criteria, there were 464 stillbirths, for a stillbirth rate of 7.2/1,000 total births. Descriptive characteristics of the entire cohort excluding fetal anomalies and aneuploidy are located in Table 1. Body mass index (BMI) was the least robust variable in both cohorts with less than 10% missing values. These populations were diverse in terms of demographic characteristics. Fetal anomalies or aneuploidy affected 6,487 pregnancies accounting for 10.7% of the population. There were 464 (0.72%) stillbirths in the cohort including fetal anomalies and aneuploidy and 330 (0.58%) stillbirths in the cohort excluding anomalies and aneuploidy for a difference of 134 stillbirths or 29%. Descriptive characteristics of the cohort including anomalies and aneuploidy can be further explored in S1 Table.

**Table 1:** Maternal demographic and pregnancy characteristics among pregnancies excluding anomalies or aneuploidy.

Characteristic	N = 57,326
Maternal age*	31 (26,35)
Maternal age <19 n(%)	3,131 (5.46)
Maternal age ≥40 n(%)	3,272 (5.71)
Parity*	1 (0,2)
Nulliparous n(%)	22,121 (38.59)
**Race**	
Black n(%)	13,048 (22.76)
White n(%)	35,383 (61.72)
Other n(%)	8,895 (15.52)
**Maternal BMI kg/m**^**2**^(n = 51,669)*	25.23 (22.36, 30.02)
Underweight n(%)< 18.5	871 (1.69)
Normal Weight n(%)18.5–24.9	23,941 (46.33)
Overweight n(%)25-29.9	13,814 (26.74)
Class I Obesity n(%)30-34.9	6,879 (13.31)
Class II Obesity n(%)35-39.9	3,422 (6.62)
Class III Obesity n(%)≥40	2,742 (5.31)
**Current smoker n(%)**	6,265 (10.96)
**Chronic hypertension (n = 57,326) n(%)**	1,430 (2.49)
**Preeclampsia n(%)**	4,587 (8.08)
**Pre-gestational diabetes (n = 57,326)n(%)**	1,112 (1.94)
**Gestational diabetes n(%)**	3,020 (5.32)
**Stillbirth**	330 (0.58)

*Median (interquartile range)

The associations between stillbirth and each candidate predictor are summarized in Table 2. The unadjusted ORs for each individual risk factor varied with fetal anomalies and aneuploidy having the greatest ORs for stillbirth 3.44 (95% CI 2.82–4.22) (S2 Table). When fetal anomalies and aneuploidy were excluded, only black race compared to all other races (OR 2.39 95% CI 1.92–2.98), class III obesity compared to BMI < 25 kg/m^2^ (OR 2.52 95% CI 1.72–3.69), chronic hypertension (CHTN) (OR 2.27 95% CI 1.41–3.66) and pre-gestational diabetes (OR 2.26 95% CI 1.32–3.87) demonstrated ORs greater than 2.0 with 95% CI that did not cross unity (Table 2).

**Table 2:** Unadjusted odds ratios for stillbirth excluding anomalies or aneuploidy.

Characteristic	OR (95% CI) n = 57,326
**Maternal age**	
Categorical	
≤ 18	1.71 (1.07–2.74)
19–34	Ref
35–39	0.87 (0.66–1.14)
40–44	0.88 (0.53–1.46)
≥ 45	2.44 (0.60–9.91)
Dichotomous	
<19	1.55 (1.04–2.30)
≥35	0.92 (0.71–1.20)
≥40	0.95 (0.59–1.53)
≥ 45	2.48 (0.61–10.04)
**Nulliparity**	1.25 (1.00–1.55)
**Race**	
Black	2.39 (1.92–2.98)
White	0.51 (0.41–0.64)
Other	0.84 (0.61–1.15)
**Maternal BMI kg/m**^**2**^	
Categorical	
< 25	Ref
25–29.9	0.89 (0.65–1.21)
30–34.9	1.47 (1.06–2.04)
35–39.9	1.36 (0.87–2.12)
≥40	2.52 (1.72–3.69)
Dichotomous	
> 25	1.26 (1.00–1.60)
> 30	1.73 (1.36–2.19)
> 40	2.38 (1.66–3.41)
**Current smoker**	1.50 (1.00–2.24)
**Chronic hypertension**	2.27 (1.41–3.66)
**Pre-gestational diabetes**	2.26 (1.32–3.87)

The initial models to predict stillbirth at each gestational age included maternal age, BMI, nulliparity, black race, current smoker, chronic hypertension, pre-gestational diabetes. Fetal anomalies and aneuploidy were included as a risk factor in the initial models developed out of the cohort which included these patients. The model with the greatest AUC was the model which predicted stillbirth at or beyond 24 weeks among the cohort that included anomalies and aneuploidy (AUC 0.70 95% CI 0.67–0.74) (S3 Table).

The model with the best performance for the prediction of stillbirth at or beyond 32 weeks excluding anomalies and aneuploidy is demonstrated in Table 3. After evaluating model discrimination using the area under the curve, model calibration and the IDI, the final model included maternal age as a categorical variable (≤ 18 years, 19–34 years, 35–39 years, and ≥ 40 years), black race, nulliparity, BMI as a categorical variable (BMI < 25 kg/m^2^, BMI 25–29.9 kg/m^2^, 30–34.9 kg/m^2^, 35–39.9 kg/m^2^, and ≥ 40 kg/m^2^), current smoker, CHTN and pre-gestational diabetes. Model discrimination is modest with an AUC of 0.66 (95% CI 0.60–0.72). The statistical cutpoint as determined by the methods of Youden and Liu were 18 stillbirths /10,000 ongoing pregnancies at 32 weeks gestation, with a sensitivity of 59% and specificity of 65%.

**Table 3:** Final model for the prediction of stillbirth at or beyond 32 weeks gestation excluding fetal anomalies and aneuploidy^a^.

Risk factor	OR	B-coefficient	p-value
Maternal Age (years)			
≤18	0.42	-.8707	.23
19–34	Ref	Ref	Ref
35–39	1.24	.2094	.41
≥40	1.55	.4377	.28
Black	2.35	.8536	.00
Nulliparity	1.41	.3423	.11
Maternal BMI (kg/m^2^)			
BMI< 25	Ref	Ref	Ref
BMI 25–29.9	0.98	-.0219	.94
BMI 30–34.9	1.75	.5607	.04
BMI 35–39.9	0.55	-.5948	.27
BMI ≥ 40	1.17	.1593	.70
Smoking	1.25	.2270	.45
CHTN	1.87	.6255	.16
Pre-gestational diabetes	2.68	.9863	.03
constant	-6.8772		

^a^AUC: 0.66 95% CI 0.60–0.72

Although statistical cutpoints serve as a helpful tool, the goal is to mathematically maximize sensitivity and specificity which may not be the most clinically relevant approach for assessing clinical utility. Therefore, to determine a clinical cutpoint, we used a clinical scenario to model a patient that would routinely receive antenatal testing under our current antenatal testing protocol: for a 25 year old white multiparous female with a BMI of 24 kg/m^2^ with pre-gestational diabetes who does not smoke and does not have chronic hypertension the risk of stillbirth is 27/10,000 ongoing pregnancies at 32 weeks. Table 4 illustrates model performance at various probabilities of stillbirth including the statistical and clinical cut-points described above, ranging from a risk of 5 stillbirths/10,000 ongoing pregnancies (100% sensitivity, 0.11% specificity, positive likelihood ratio (+LR) of 1.0) to 73 stillbirths/10,000 ongoing pregnancies (4.2% sensitivity, 99.1% specificity, +LR of 4.37). The calculator developed based on the formula: *Probability = e^logit/1+e^logit where logit = -6*.*8772–0*.8707*Maternal age < 18 + 0.2094*maternal age 35–39 + 0.4377*maternal age > 40 + 0.8536*black race + 0.3423*nulliparity–.0219*BMI 25–29.*9 + 0*.5607*BMI 30–34.*9–0*.5948*BMI 35–39.*9 + 0*.1593*BMI >40 + 0.2770*Currently_smoking+0.6255*CHTN + 0.9863*pre-gestational diabetes, can be found in a user friendly format at https://www.dropbox.com/s/x4hwurqehhdc2a4/FINAL%20SB%20calculator.xlsx?dl=0 or S1 File.

**Table 4:** Clinical performance of the stillbirth calculator for the prediction of stillbirth at or beyond 32 weeks gestation excluding fetal anomalies and aneuploidy^a^.

Cutpoint: number of stillbirths/10,000 ongoing pregnancies	Sensitivity %	Specificity %	Correctly Classified	+LR
5	100	.11	.30	1.00
12	87.5	23.6	23.7	1.14
17	60.4	62.8	62.8	1.62
18	55.2	67.4	67.4	1.69
27	33.3	83.7	83.6	2.04
34	25.0	91.7	91.6	3.03
73	4.2	99.1	98.9	4.37

^a^Probability(risk) of stillbirth *= e^logit/1+e^logit where logit = -6*.*8772–0*.8707*Maternal age < 18 + 0.2094*maternal age 35–39 + 0.4377*maternal age > 40 + 0.8536*black race + 0.3423*nulliparity–.0219*BMI 25–29.*9 + 0*.5607*BMI 30–34.*9–0*.5948*BMI 35–39.*9 + 0*.1593*BMI >40 + 0.2770*Currently_smoking+0.6255*CHTN + 0.9863*pre-gestational diabetes

The clinical risk score to predict stillbirth at or beyond 32 weeks is located in Table 5. Each weight was determined by rounding the OR from the final risk prediction model to the nearest whole number, with a weight of 0 assigned to the referent group. The scores in the dataset ranged from 0 to twelve with a median of 2 and an interquartile range of 1–3. The risk score and clinical performance at each point cut-point is demonstrated in Table 6. The statistical cutpoint as determined by the methods of Youden and Liu were determined to be 1.5 points and 2.5 points, respectively. The clinical cutpoint as determined by the scenario of a 25 year old white multiparous female with a BMI of 24 kg/m^2^ with pre-gestational diabetes who does not smoke and does not have chronic hypertension is 3 points. Similar to the model using the beta coefficients, the risk score demonstrated modest discrimination (AUC 0.64 96% CI 0.58–0.70) which was not significantly different from the more complicated calculator based model (p = 0.25). Additionally, the clinical performance of the simple risk score did not demonstrate a significant difference in sensitivity from the calculator based model as determined by the IDI comparing the two models (p = 0.69).

**Table 5:** Stillbirth risk score for the prediction stillbirth at or beyond 32 weeks gestation excluding fetal anomalies and aneuploidy^a^.

Risk factor	OR	Points assigned
**Maternal Age (years)**^b^		
≤18	0.42	1
19–34	Ref	0
35–39	1.24	1
≥40	1.55	2
**Black**	2.35	2
**Nulliparity**	1.41	1
**Maternal BMI (kg/m**^**2**^**)**^a^		
< 25	Ref	0
25–29.9	0.98	1
30–34.9	1.75	2
35–39.9	0.55	1
≥ 40	1.17	1
**Smoking**	1.25	1
**Chronic hypertension**	1.87	2
**Pre-gestational diabetes**	2.68	3
**Total Score Possible**		**13**

^a^ AUC 0.64 95% CI 0.58–0.70

^b^Categories with mutually exclusive points, the greatest score for either age or BMI is 2

**Table 6:** Clinical performance of the stillbirth risk score to predict stillbirth at or beyond 32 weeks excluding fetal anomalies and aneuploidy.

Score cut-point	Sensitivity %	Specificity %	Correctly Classified %	+LR
**0**	100	0	0.2	1.0
**1**	91.7	12.1	12.2	1.04
**2**	78.1	40.9	41.0	1.32
**3**	53.1	65.4	65.3	1.54
**4**	34.4	82.6	82.5	1.98
**5**	22.9	93.1	93.0	3.32
**6**	11.5	97.5	97.4	4.64
**7**	3.13	99.1	98.9	3.48
**8**	0	99.6	99.4	0
**9**	0	99.9	99.7	0
**10**	0	100	99.8	0
**11**	0	100	99.8	0

Stratification by gestational age beyond 32 weeks did not have a significant impact on model discrimination or on individual stillbirth risk prediction. There was no difference in the predictive accuracy of the models to predict stillbirth beyond 32, 34 or 36 weeks, (AUC_32_ 0.6438 95% CI (0.572–0.716), AUC_34_ 0. 0.6473 95% CI (0.575–0.719) AUC_36_ 0.6508 95% CI (0.577–0.725) p = 0.81), respectively. Furthermore, when the model was used to predict individual risk based on presence or absence of risk factors, there was no difference in stillbirth risk. For example, a nulliparous woman with class III obesity and chronic hypertension is estimated to have a stillbirth risk of 31.7/10,000 at or beyond 32 weeks, 34 weeks and 36 weeks. Further stratification of stillbirth risk beyond 37 weeks was not feasible due to sample size limitations.

## Discussion

### Main findings

Using maternal risk factors, we were able to develop and internally validate a stillbirth risk calculator and a simplified stillbirth risk score to predict the risk of stillbirth at or beyond 32 weeks gestation. We determined both statistical and clinical cut-points which could be applied clinically as an evidence based approach to identify women who would benefit most from antenatal testing for the prevention of stillbirth.

### Strengths and limitations

One of the major strengths of our study is that to the authors’ knowledge, it is the first clinical prediction tool developed to predict stillbirth. At present, the only proven intervention to prevent stillbirth is delivery; however, antenatal testing has been used for several decades and has become standard of care in the U.S. for the monitoring of the fetus deemed to be at increased risk for stillbirth. Therefore, in-line with current ACOG guidelines for the initiation of antenatal testing designed to reduce the risk of stillbirth, we developed a model to predict stillbirth at or beyond 32 weeks, when the initiation of antenatal testing would be clinically appropriate. Additionally, our clinical aim also propelled us to develop a model that can be applied at a routine prenatal visit. Although the inherent limitations of sample size may have impacted our ability to evaluate many risk factors, particularly in light of the relatively rare outcome, stillbirth, our sample size afforded us the ability to combine multiple common clinical risk factors. Our user-friendly stillbirth calculator was developed for use in an office setting with the aid of a smartphone application. As an alternative, we developed a simplified stillbirth score which does not compromise performance significantly and can easily be tallied without the use of an electronic device. Finally, our rich database of patient level data allowed us to incorporate maternal risk factors without the use of statistical manipulation to account for missing data.

Our study was not without limitation. First, it should be noted that this is not a study on the natural history of stillbirth, and quantifying the impact on stillbirth of obstetric management such as antenatal testing or early delivery is not possible. However, given the current standard of care in the U.S. which involves initiating antenatal testing when the stillbirth risk is assessed as being sufficiently high, our study is generalizable to current obstetric practice. Additionally, the outpatient antenatal testing protocol used at Washington University in St. Louis would be expected to provide consistency in management. Second, when BMI was incorporated into a multivariate model to predict stillbirth at or beyond 32 weeks, the expected trend toward increasing risk of stillbirth with increasing BMI was no longer observed. [16, 17] However, we did see this trend in our univariate analysis and when we examined the final model that predicts stillbirth at or beyond 20 weeks (Table 2). This would suggest the timing of stillbirth may be earlier for obese women. One possible explanation is that with increased incidence of medical and obstetric comorbidities in these patients, the later stillbirths are prevented due to increased need for intervention at an earlier gestational age. Third, given that patients and providers may be less inclined to report a negative outcome such as stillbirth, excluded cases could be a source of selection bias. However, sensitivity analyses were performed and do not support significant impact of such potential bias on the study results. Finally, the model used to develop the stillbirth risk calculator and stillbirth risk score is less parsimonious than we would like for a clinical tool. However, we used a rigorous methodical approach to model development as described. Although a certain level of compromise in model performance is generally tolerable when developing a clinical prediction tool, we felt that the performance of the model was limited at baseline and therefore compromising further performance for a more parsimonious model would not be tolerable in this case.

### Interpretation

Regarding the clinical accuracy of the models, the discrimination as determined by the AUC was better than chance, but was still relatively low. However, it has been demonstrated that any model developed to predict a rare outcome using covariates that have individual risks that are relatively low for the outcome would be expected to have low discriminative accuracy. One such example is the Gail model, developed in 1989 and utilized by clinicians and researchers around the world to predict the risk of breast cancer, determine trial eligibility and aid in clinical decision making regarding treatment options[18]. The original Gail model was modified in 1999 and used for eligibility in the *Breast Cancer Prevention Trial*[19]. Rockhill and colleagues examined the clinical performance of the modified Gail model and found the discriminative accuracy to be modest at best with an AUC Of 0.58 (95%CI 0.56–0.60)[20].

Previous studies have investigated maternal uterine artery Dopplers studies, serum markers and fetal biometric parameters in an effort to predict stillbirth related to impaired placentation, but investigation of maternal factors has been limited until recently[21–23]. In 2016 Yerlikya and colleagues demonstrated maternal risk factors identified early in pregnancy could predict one third of stillbirths with model discrimination very similar to our findings (AUC 0.642 95% CI 0.612–0.672)[24]. In a separate publication later in 2016, the group incorportated maternal factors into their previous modeling efforts evaluating uterine artery Doppler studies and fetal biometry. In this study, Akolekar et al. noted predictive accuracy of their model was again similar to our model discrimination (AUC 0.652 95% CI 0.617–0.688)[25]. The authors estimated 60% of stillbirths are related to placental impairment and based on their modeling they could predict 75% of these stillbirths. However, this did not include the 40% of stillbirths resulting from causes other than placental impairment. Furthermore, the aim of these investigations was to evaluate stillbirth prediction early in pregnancy and although the authors did evaluate stillbirth prediction at various gestational ages, the models calculated stillbirth risk retrospectively from the selected gestational age cutpoint including gestational ages when antepartum surveillance would not be possible or clinically appropriate as a potential intervention. Yerlikaya and colleagues did investigate the prediction of stillbirth beyond the early term period starting at 37 weeks, but found the model to have low predictive accuracy (AUC 0.581 95% CI 0.495–0.666 and AUC 95% CI) [24]. The recent investigations by this group highlight the importance of incorporating maternal factors into stillbirth risk prediction modeling efforts and is an important contribution to the field for planning future investigation. Our study differs significantly from the previous investigations primarily by our clinical aim to provide an evidence based approach to the estimation of stillbirth risk which can be utilized even in a resource poor setting. As such, our goal was to identify women who may benefit most from antenatal testing; therefore, we modeled stillbirth risk prospectively to estimate the risk of stillbirth starting at a gestational age cutpoint (i.e. 32 weeks) to then give the risk of stillbirth going forward through the remainder of gestation.

To illustrate the clinical application of our tools we use the clinical cut-points which set the threshold for initiating antenatal testing at the level of a woman who would routinely receive antenatal testing in the U.S., that of an otherwise healthy white multiparous pre-gestational diabetic. Using the stillbirth calculator and a screen positive stillbirth risk of 27/10,000, based on the sensitivity and specificity of the stillbirth calculator at this cut-point, 1,671/10,000 women will screen positive, of which, 9 stillbirths will occur. If we then use information from the largest study published on the use of antepartum fetal heart rate monitoring which demonstrated 1.9/1,000 stillbirths occurred within one week of a reactive non-stress test (NST), and subtract this from the baseline stillbirth risk of our population of 6/1,000 (excluding fetal anomalies and aneuploidy), then we deduce that approximately 4/1,000 stillbirths are prevented with the use of NSTs or NSTs can prevent two-thirds of stillbirths[26]. Therefore, the number needed to test to prevent one stillbirth is 279. If we apply the same logic to the stillbirth score, then at a cut-point of 3 points, 3,468/10,000 women will screen positive and 12 stillbirths will occur, of which 8 could be prevented by NSTs, for a final number needed to test to prevent a single stillbirth of 434. Therefore, if we use the stillbirth score, we will prevent an additional two stillbirths over the stillbirth calculator at the expense of performing antenatal testing on an additional 310 women. Furthermore, in 2006, Reddy et al. identified increasing rates of stillbirth with advancing gestational age starting at 32 weeks which suggests the impact of our stillbirth prediction beyond 32 weeks may account for the largest proportion of stillbirths that occur annually which may have an even greater impact on reducing overall national stillbirth rates[14].

To perform a very rudimentary cost analysis, we over simplify and assume the value of an NST is equal to what private insurers are willing to pay, approximately $75 on average as of 2016. At Washington University the antenatal testing protocol includes twice weekly NSTs. Therefore, if we start NSTs at 32 weeks and assume delivery at 39 weeks then the total cost of antenatal testing is $1,050 for a cost difference of $162,750 per stillbirth avoided. Although a formal cost-effectiveness analysis would be required to fully analyze the costs and benefits of performing large volumes of additional antenatal testing for the benefit of a few stillbirths, considering the gravity of the outcome at stake, the additional cost of using either the stillbirth calculator or the stillbirth score to determine candidacy for antenatal testing is likely outweighed by the benefit of preventing 6 to 8 additional stillbirths per 10,000 ongoing pregnancies.

### Conclusion

In conclusion, accurate prediction of stillbirth at this point in time is difficult. However, using a rigorous methodical approach to model development and a thorough exploration of clinical performance, we have developed a clinical prediction tool to predict stillbirth at or beyond 32 weeks as an evidenced based approach to the initiation of antenatal testing to reduce stillbirths. Prior to recommending the use of any risk prediction tool into routine clinical practice, the most important next step is external validation[27]. We propose that our modeling efforts can serve as the basis for future independent validation studies. Through our modeling, we project that initiating antenatal testing with a stillbirth score of 3 or more will reduce the stillbirth rate by 8/10,000 ongoing pregnancies, from a national average of 6.2/1,000 births to 5.4/1,000 births, a difference that seems small, but when put into perspective, would accomplish the Healthy People 2020 goal for stillbirth reduction[2].

## Supporting information

S1 Table Maternal demographic and pregnancy characteristics of the entire cohort among pregnancies including fetal anomalies and aneuploidy. *Median (interquartile range).
(DOCX)

S2 Table Unadjusted odds ratios for stillbirth including anomalies and aneuploidy. (DOCX)

S3 Table Model for stillbirth ≥ 24 weeks^a^. ^a^AUC: 0.703 95% CI 0.668–0.737.
(DOCX)

S1 File Interactive stillbirth calculator. (XLSX)
