# Supplementary material for: A stillbirth calculator: Development and internal validation of a clinical prediction model to quantify stillbirth risk
Source: PLoS One. 2017 Mar 7;12(3):e0173461. doi: 10.1371/journal.pone.0173461 (PMC5340400; doi:10.1371/journal.pone.0173461)
Supplement: S1 Table — *Median (interquartile range). (DOCX) [file pone.0173461.s001.docx]

S1 Table: Maternal demographic and pregnancy characteristics of the entire cohort among pregnancies including fetal anomalies and aneuploidy

| **Characteristic** | N=64,173 |
| --- | --- |
| Maternal age* | 30 (26, 35) |
| Maternal age <19 n(%) | 3,658 (5.70) |
| Maternal age >40 n(%) | 3,601 (5.61) |
| **Parity*** | 1 (0,2) |
| Nulliparous n(%) | 24,895 (38.79) |
| **Race** |  |
| Black n(%) | 14,724 (22.94) |
| White n(%) | 39,517 (61.58) |
| Other n(%) | 9,932 (15.48) |
| **Maternal BMI*(n=57,380)** | 25.16 (22.35, 29.90) |
| Underweight n(%)  < 18.5 | 1,014 (1.77) |
| Normal Weight n(%)  18.5- 24.9 | 26,816 (46.73) |
| Overweight n(%)  25-29.9 | 15,443 (26.91) |
| Class I Obesity n(%)  30-34.9 | 7,552 (13.16) |
| Class II Obesity n(%)  35-39.9 | 3,679 (6.41) |
| Class III Obesity n(%)  >40 | 2,876 (5.01) |
| **Current smoker (n=63,957) n(%)** | 7,119 (11.13) |
| **Chronic hypertension n(%)** | 1,550 (2.42) |
| **Preeclampsia (n=63,567 ) n(%)** | 5,091 (8.01) |
| **Pre-gestational diabetes n(%)** | 1,208 (1.88) |
| **Gestational diabetes (n=63,567) n(%)** | 3,233 (5.09) |
| **Fetal anomaly or aneuploidy n(%)** | 6,847 (10.67) |
| **Stillbirth n(%)** | 464 (0.72) |

*Median (interquartile range)
