# Supplementary material for: A stillbirth calculator: Development and internal validation of a clinical prediction model to quantify stillbirth risk
Source: PLoS One. 2017 Mar 7;12(3):e0173461. doi: 10.1371/journal.pone.0173461 (PMC5340400; doi:10.1371/journal.pone.0173461)
Supplement: S2 Table — (DOCX) [file pone.0173461.s002.docx]

S2. Supplement Table 2: Unadjusted odds ratios for stillbirth including anomalies and aneuploidy

| **Characteristic** | **OR (95% CI)**  **n=64,173** |
| --- | --- |
| **Maternal age** |  |
| Categorical |  |
| < 18 | 1.48 (0.98-2.22) |
| 19-34 | Ref |
| 35-39 | 0.78 (0.62-0.99) |
| 40-44 | 0.76 (0.48-1.20) |
| > 45 | 1.76 (0.43-7.14) |
| Dichotomous |  |
| <19 | 1.48 (1.06-2.07) |
| >35 | 0.81 (0.64-1.019) |
| >40 | 0.84 (0.54-1.28) |
| > 45 | 1.85 (0.46-7.47) |
| **Nulliparity** | 1.29 (1.18-1.79) |
| **Race** |  |
| Black | 2.10 (1.74-2.53) |
| White | 0.61 (0.50-0.72) |
| Other | 0.76 (0.58-1.01) |
| **Maternal BMI kg/m^2^** |  |
| Categorical |  |
| < 25 | Ref |
| 25-29.9 | 0.91 (0.71-1.16) |
| 30-34.9 | 1.24 (0.93-1.65) |
| 35-39.9 | 1.23 (0.83-1.81) |
| >40 | 2.09 (1.48-2.95) |
| Dichotomous |  |
| > 25 | 1.15 (0.94-1.39) |
| > 30 | 1.46 (1.18-1.79) |
| > 40 | 2.05 (1.48-2.85) |
| **Current smoker** | 1.50 (1.08-2.10) |
| **Chronic hypertension** | 2.03 (1.32-3.11) |
| **Pre-gestational diabetes** | 2.00 (1.23-3.25) |
| **Fetal anomaly or aneuploidy** | 3.44 (2.82-4.22) |
