# Supplementary material for: A stillbirth calculator: Development and internal validation of a clinical prediction model to quantify stillbirth risk
Source: PLoS One. 2017 Mar 7;12(3):e0173461. doi: 10.1371/journal.pone.0173461 (PMC5340400; doi:10.1371/journal.pone.0173461)
Supplement: S3 Table — aAUC: 0.703 95% CI 0.668–0.737. (DOCX) [file pone.0173461.s003.docx]

**S3. Supplement Table 3: Model for stillbirth > 24 weeks^a^**

| **Risk factor** | **OR** | **Β-coefficient** | **p-value** |
| --- | --- | --- | --- |
| Age > 40 (y/n) | 1.141 | .1318 | .636 |
| Black (y/n) | 1.891 | .6371 | .000 |
| Nulliparity (y/n) | 1.236 | .2122 | .106 |
| Maternal BMI (kg/m^2^) | 1.006 | .0055 | .105 |
| Current smoker (y/n) | 1.420 | .3506 | .049 |
| Chronic hypertension (y/n) | 2.176 | .7774 | .005 |
| Pre-gestational diabetes (y/n) | 2.632 | .9676 | .001 |
| Anomaly or aneuploidy (y/n) | 4.186 | 1.4318 | .000 |

^a^AUC: 0.703 95% CI 0.668-0.737
